# Eprinomectin nanoemulgel for transdermal delivery against endoparasites and ectoparasites: preparation, *in vitro* and *in vivo* evaluation

**DOI:** 10.1080/10717544.2019.1682720

**Published:** 2019-11-18

**Authors:** Yujuan Mao, Xiaolan Chen, Bohui Xu, Yan Shen, Zixuan Ye, Birendra Chaurasiya, Li Liu, Yi Li, Xiaoling Xing, Daquan Chen

**Affiliations:** a Jiangsu Animal Husbandry and Veterinary College, Taizhou, China;; b School of Pharmacy, Nantong University, Nantong, China;; c Department of Pharmaceutics, School of Pharmacy, China Pharmaceutical University, Nanjing, China;; d School of Pharmacy, Yantai University, Yantai, China

**Keywords:** Bioadhesion, eprinomectin, nanoemulgel, transdermal delivery system, transdermal permeability

## Abstract

Nanoemulgels are composed of O/W nanoemulsion and hydrogels and are considered as ideal carriers for the transdermal drug delivery because these have high affinity to load hydrophobic drugs. The stable formulation of eprinomectin (EPR) is very challenging because of it is high hydrophobic nature. In this work, we have prepared EPR loaded nanoemulgel for the treatment of endo- and ectoparasites. The surface morphology of optimized formulations was characterized by scanning electron microscopy. Additionally, skin permeability and irritation tests were conducted for *in vitro* safety and *in vivo* skin retention and pearmeation test of EPR nanoemulgel were conducted for efficacy study. Obtained results indicated that the optimized formulation had good shear-thinning behavior, bioadhesiveness properties, and are nanosized droplets with porous internal structure, which are required for topical application. Furthermore, this formulation has showed good skin permeability in comparison to suspension and has no skin irritating property. Overall, the obtained results proved that nanoemulgel is a promising carrier for transdermal drug delivery and EPR nanoemulgel is a promising formulation for the treatment of endo- and ectoparasites.

## Introduction

Hydrogels, for its three-dimension cross-linked network structure, are well known as excellent carriers for drug-loading. The mechanism of drug release from the matrix is determined by the physicochemical properties of the hydrogel and the method of drug-loading. For example, swelling-controlled release mechanism always occurs in the process of small molecule drugs release from HPMC tablets (Lin & Metters, 2006). Additionally, hydrogels have good rheological and bio-adhesive property and are biocompatible, with these unique characteristic features, hydrogels are consider as a better carrier for topical drug delivery (Gao et al., 2016).

However, the hydrophilicity of hydrogel limits the applications for the delivery of hydrophobic drugs. To overcome this shortfall, a novel transdermal delivery system termed emulgel was developed, where emulsion was thickened by hydrogel. Hydrophobic drugs were loaded in the oil cores and the droplets of emulsion are entrapped into the hydrogel cross-linked network. Upon application, loaded drug in the internal phase pass through the external phase to the skin and slowly get absorbed (Ajazuddin et al., 2013). In order to improve the retention ability, bioadhesive polymers such as carbomer 934, carbomer 940, hydroxypropylmethylcellulose, are used as gel forming agents, which can influence the bioadhesive behavior of emulgel (Djekic et al., 2019). The drug release pattern and quantity from this emulgels are influenced by the types of gelling agents, concentration of emulsifier and oil base used in emulsion (Mohamed, 2004). By controlling emulsion bases in emulgels, the bioadhesive properties and sustained drug release pattern from emulgels can be controlled for prolonged therapeutic effect at desired site of action (Palcso et al., 2019).

The viscosity of emulgels is lower in comparison to hydrogels which makes it easy to spread at the site of application. Gel forming agents in hydrogels exhibit multiple functions such as thickeners (increase the viscosity of formulation) and as emulsifiers (decrease the interfacial tension) to enhance the stability of emulgels (Bonacucina et al., 2009; Javed et al., 2018; Sohail et al., 2018). Thus, emulgels have been researched for the delivery of hydrophobic drug such as propolis, cyclosporine A, and amphotericin B (Shen et al., 2015; Balata et al., 2018; Pinheiro et al., 2019). From various experimental finding regarding emulgel delivery system, it was found that nanosized (generally range from 20 to 500 nm) emulsion gained the advantages of large surface area which allows rapid penetration through the pores to reach the systemic circulation. Drug from nanoemulgel can permeate the skin through both transcellular and paracellular route, while nanoemulsion deliver the drug through transcellular route only (Rambharose et al., 2017; Sengupta & Chatterjee, 2017).

It has been found that parasites always have negative effects on the growth and fertility of domestic animals. For instance, the heartworm disease in cats may present with clinical signs such as coughing, vomiting, dyspnea or severe respiratory distress, and gastrointestinal nematode infections in cows would also cause the loss of milk production and reduction of quality (May et al., 2017). The worse thing is that, for the close contact between human and domestic animals, humans would be easily infected, which poses a threat to public’s health (Tielemans et al., 2014). Eprinomectin (EPR; 4″-epi-acetylamino-4″-deoxy-avermectin B1) is a hydrophobic 16-membered macrocyclic lactone developed by Merck research laboratories. Other than its great therapeutic efficacy against endo-and ectoparasites such as gastrointestinal nematodes, horn fly, and lice (Shoop et al., 1996; Cringoli et al., 2003), it was proved to be safe to lactating dairy animals (Baoliang et al., 2006). The marketed EPR formulation includes topical formulation (EPRINEX^®^ Pour-on, Merial) and injectable formulation (Eprecis^®^ 20 mg/mL, Ceva) (Hamel et al., 2018; Termatzidou et al., 2019). However, the Pour-on formulation has weak adhesion and it is administered by pouring along the animals’ back line (Hamel et al., 2018), which may lead to short skin contact time and decrease of efficacy. Injectable formulation could only be used by technicians who have been trained professionally.

To meet the requirements of long-term treatment and topical use, we have developed EPR nanoemulgel consisting of nanoemulsion and hydrogel with good bioadhension and better permeability than conventional emulgel and suspension.

## Materials and methods

### Materials and animals

EPR (*B*_1a_ + *B*_1b_ ≥ 95%, *B*_1a_ ≥ 90%) was purchased from North China Pharmaceutical Group Aino Co., Ltd (Shijiazhuang, China). Tween 80 (CP) and ethanol was from Nanjing Chemical Reagent Co., Ltd (Nanjing, China). Sodium hydroxide was obtained from Sinopharm Chemical Reagent Co., Ltd (Shanghai, China). Carbomer 940-1 was provided from Nanjing Weier Pharmaceutical Co., Ltd (Nanjing, China). Castor oil (CP) was purchased from Shanghai Lingfeng Chemical Reagent Co., Ltd (Shanghai, China). Caprylocaproyl Macrogolglycerides (Labrasol^®^) was purchased from Gattefossé Corporation (Lyon, France). Acetonitrile (high performance liquid chromatography [HPLC] grade, 99.9%) was supplied by Tedia Chemical (Fairfield, OH). Fluorescein isothiocyante and 4% polyformaldehyde were obtained from ShanghaiYuanye Bio-Technology Co., Ltd (Shanghai, China).

L929 mouse fibroblasts were obtained from Cell Bank of Chinese Academy of Sciences (Beijing, China). Animals were purchased from the Experimental Animal Center of Nanjing Qinglongshan (Nanjing, China). All animal experiments were carried out in accordance with the National Institute of Health Guide for the Care and Use of Laboratory Animals and approved by the Animal Ethics Committee of China Pharmaceutical University.

### Methods

#### Preparation of EPR nanoemulsion

**Figure 1.**
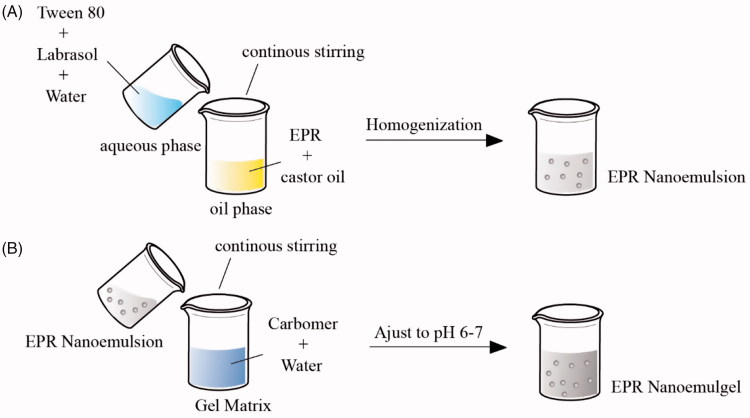
Schematic representation of preparation of EPR nanoemulsion (A) and EPR nanoemulgel (B).

To prepare EPR nanoemulsion, first non-aqueous phase was prepared by dissolving 0.55 g EPR into 1 mL of ethanol with the help of ultrasonic bath followed by mixing it with castor oil in 40 °C water bath. Then after, mixture was placed into suitable sized round bottom flask and was fixed on rotary evaporating at 38 °C (RE-52AA, Shanghai Yarong Biochemistry Instrument Factory, Shanghai, China) for 20 min to remove the ethanol. Second, 10 g aqueous phase was prepared by mixing (0.22, 0.33, and 0.44 g) Tween 80 and (0.22, 0.33, and 0.44 g) Labrasol^®^ into water. Then, the aqueous phase was slowly added into non-aqueous phase with continuous stirring on magnetic stirrer (85-2, Shanghai Sile Instrument Co., Ltd, Shanghai, China) followed by homogenization at 8000 r/min (XHF-D, Ningbo Scientz Biotechnology Co., Ltd, Ningbo, China) for 6 min to get the primary emulsion. Further, this primary emulsion was passed though high-pressure homogenizer at 350 bar for three cycles (AMH-3, ATS Nano Technology Co., Ltd, Suzhou, China) to get the final O/W EPR nanoemulsion (Figure 1(A)).

#### Preparation of EPR nanoemulgel

To prepare EPR nanoemulgel, first hydrogel matrix was prepared by soaking (0.05, 0.1, and 0.2 g) carbomer 940 into water to form 10 g carbomer gel matrix. Then after, 1 g EPR nanoemulsion (EPR 0.05 g) was added to the gel matrix with slow continuous stirring. The final EPR nanoemulgel was obtained by adjusting pH to 6–7 with the addition of sodium hydroxide aqueous solution (Figure 1(B)). At the same time, the hydrogel incorporated with EPR emulsion was EPR emulgel.

#### Optimization of EPR nanoemulgel

Each composition in the formulation should be studied in order to investigate their individual effects on the EPR formulation (such as viscosity of nanoemulgel and *Ke* of nanoemulsion). Thus, the effect of castor oil (0.40, 1.19, and 2.00 g), Tween 80 (0.22, 0.33, and 0.44 g), and Labrasol^®^ (0.22, 0.33, and 0.44 g) was studied. Effect of one composition at one time was studied.

#### Stability test (determination of *Ke*)

The stability test of the prepared emulsion was performed by centrifugation method, which is important for the homogenous nanoemulgel (Mouri et al., 2016). For this, 5 mL of the nanoemulsion was placed in a 10 mL centrifuge tube and was centrifuged at 4000 r/min (KDC-140HR, Anhui USTC Zonkia Scientific Instrument Co., Ltd, Hefei, China) for 15 min. After centrifugation, about 25 μL of samples from the bottom of the test tube was taken out accurately by a micro-pipette (50 μL) and diluted to 10 mL with distilled water. Distilled water was adopted as a blank to measure the absorbance at a visible wavelength of 500 nm. A sample of 25 μL from uncentrifuged nanoemulsion was obtained by the same method.

The stability constant *Ke* was calculated as Equation (1):

(1) $$Ke=\frac{A_{0}-A}{A}\;$$

where, *A*_0_ was the absorbance of the diluted nanoemulsion without centrifugation at the set wavelength; *A* was the absorbance of the diluted nanoemulsion after centrifugation at the same wavelength.

Due to the difference in density between the oil and aqueous phases, the oil droplets will float during the centrifugation process, which lead to change of absorbance before and after centrifugation. In this case, smaller stability constant *Ke* indicates nanoemulsion is more stable.

#### Rheological test

The viscosity of variable nanoemulgels was measured by a rheometer (DV-III Ultra, Brookfield, Middleboro, MA) using SC4-16 rotor at room temperature (25 °C) with the shear rate changed from 20 to 100 r/min.

#### Droplet size analysis and zeta potential

The optimized EPR nanoemulsion was diluted with water (1:1000, v/v) and the droplet size and zeta potential of diluted EPR nanoemulsion was measured by zeta sizer (Brookhaven Instruments Corporation, Holtsville, NY). EPR emulsion was characterized for zeta size and zeta potential in the same way.

#### Morphology investigation

The internal three-dimensional structures of the optimized EPR nanoemulgel, optimized blank nanoemulgel and 1% carbomer hydrogel was observed by a scanning electron microscope (SEM; Su8020, Hitachi, Tokyo, Japan). For SEM investigations, the samples were prepared by freezing rapidly in liquid nitrogen followed by freeze-drying for 48 h (Al-Abboodi et al., 2019).

#### Bioadhesion evaluation

**Figure 2.**
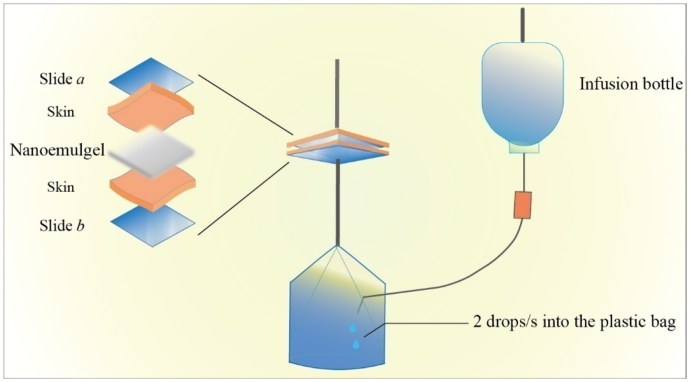
Device for the determination of bioadhension.

The adhesive property is critical for formulations which are intended to apply topically. This property can determine about the contact duration of the applied formulation on skin. This test can be evaluated by a number of test methods such as peel force test, adhesive strength test, and tack test (Al Hanbali et al., 2019). In this experiment, the bioadhesive strength of the formulations was quantified by a self-made device (Figure 2; Xiang et al., 2002).

To perform this test, healthy mice were taken and were sacrificed by cervical dislocation. Hairs from mid abdominal sides were shaved and the skin was quickly peel out to remove the subcutaneous fat and tissue. The obtained skin was preserved at –20 °C for further use.

The preserved skin was thawed at 37 °C and was cut into two pieces of 1.8 cm × 1.8 cm size and were adhered on the surfaces of slide ‘*a*’ and ‘*b*’ with the help of cyanoacrylate adhesive, respectively. Here, the EPR nanoemulgel containing 0.05, 0.1, and 0.2 g carbomer were named as nanoemulgel A, B, and C, respectively. Appropriate amount of nanoemulgel from A, B, and C with varied viscosity was weighed, respectively, and applied on the skin surface. Then after, 0.3025 N/cm^2^ pressure was applied on the slide ‘*a*’ for 10 s in order to make the two slides contact closely. As shown in Figure 2, slide ‘*a*’ was fixed and slide ‘*b*’ was attached with a plastic bag. Distilled water was dropped from infusion bottle through attached tube at the rate of 2 drops/s into the plastic bag. The detachment point of both slides was observed due to water weight into the plastic bag, once both slides started to detach, water dropping into the plastic bag was stopped and the bioadhesion force per unit area was calculated by using Equation (2) as follow:

(2) $$f=\frac{m}{r^{2}}\;$$

where, *f* represents the bioadhesion force per unit area (g/cm^2^); *m* is the total mass of water applied on slide ‘*b*’ (g); and *r* represents the size (1.8 cm) of the skin taken to apply the gel.

#### Eprinomection quantification

EPR concentration was quantify by HPLC using a LC-20AT Shimadzu system with autosampler (Shimadzu Corporation, Kyoto, Japan) and a UV detector (Shimadzu, SPD-20A UV/Vis Detector, Kyoto, Japan) at 245 nm. A Kromasil ODS column, 5 μm (250 mm × 4.6 mm) was used for separation at the column oven temperature of 30 °C. The mobile phase used was comprised of 75% acetonitrile and 25% water at the flow rate of 1 mL/min. Initially, series of known concentration of standard solutions ranging from 0.03 to 10 μg/mL were run to draw calibration curve to quantify the concentration of EPR in test samples.

#### *In vitro* permeation study

*In vitro* permeation study was conducted to evaluate the permeability of EPR from different formulations. For this, Franz diffusion cell system with a 2.27 cm^2^ effective diffusion area (TPY-2, Shanghai Huanghai Pharmaceutical Inspection Instrument Co., Ltd, Shanghai, China) was used. Skin without any defects was mounted between donor and receptor compartments in the diffusion cell, with the epidermis side facing the donor compartment. The receptor compartment was filled with a 6.5 mL receptor buffer (normal saline containing 30% methanol). After that, EPR suspension, EPR emulgel, and EPR nanoemulgel containing same quantity of EPR were put separately in the donor compartment. Then the temperature in the diffusion chamber was maintained at 37 ± 0.5 °C in a thermostatic water bath. Samples from receptor chambers were collected at predetermined time intervals (1, 2, 4, 6, 8, 10, and 12 h) with the replacement of same volume of fresh aerated receptor buffer. The solutions were filtered through membrane filter (0.22 μm) and were run on HPLC to quantify the content of EPR at each time interval.

The cumulative penetration *Q* was calculated by the following formula:

(3) $$Q_{n}=V_{0}\left(C_{n}+\sum_{1=1}^{n-1}C_{i}\right)\;$$

where, *C_n_* is the concentration of EPR in the receptor solution at the *n*th sampling point; *C_i_* is the concentration of EPR before *n*th sampling point; *V*_0_ is the volume of receptor solution (6.5 mL).

The apparent permeation coefficient (*P*_app_, cm/s) of PRZ was calculated as follows:

(4) $$P_{\mathrm{app}}=\frac{△Q}{{△t\cdot C}_{0}\cdot A\cdot 3600}$$

where, *C*_0_ is the initial EPR concentration in donor chamber; *A* is the effective diffusion area (2.27 cm^2^); △Q/△t is the slope of the straight line portion on *Q_n_* – *t* plot.

The steady-state flux (*J*_ss_, μg cm^−2^ h^−1^) was determined by:

(5) $$J_{\mathrm{SS}}=C_{0}\cdot P_{\mathrm{app}}\cdot 3600=\frac{△Q}{{△t\cdot C}_{0}\cdot A}$$

#### Cytotoxicity

The toxicity level of EPR suspension and EPR nanoemulgel was investigated by MTT assay method using L929 mouse fibroblasts cell line. Briefly, the L929 fibroblast cells were seeded into 96-well plates with cell densities of 6 × 10^3^ and incubated in RPMI 1640 with 10% FBS at 37 °C with 5% CO_2_. Both EPR suspension and EPR nanoemulgel were diluted with serum free RPMI-1460 medium in the same EPR concentration ranging from 1 to 10,000 μg/mL with the multiplication of 10. After 24 h of cell incubation, culture medium from each well was gently aspired using suction pump and test samples were added into each well. Plates were re-incubated for another 24 h in same chamber condition. After 24 h, the medium from each well was discarded and 200 μL of 0.5 mg/mL MTT solution was added into each well. Then, the media were removed and MTT formazan crystals were dissolved in 150 μL dimethyl sulfoxide. Finally, the absorbance was measured on a microplate reader at 570 nm and cell viability of each samples was calculated using Equation (6) as below:

(6) $$\text{Cell viability}\left(\%\right)=\frac{{\mathrm{OD}}_{570,\;\mathrm{sample}}}{{\mathrm{OD}}_{570,\mathrm{control}}}\times 100\%$$

#### Skin irritation test

Skin irritation test was conducted to determine the irritation safety level of the EPR nanoemulgel. In some cases, parasites would cause skin lesion (Steelman, 1976; Goldberg & Bursey, 1991), so that it is necessary to investigate if there is any irritation of preparation to the damaged skin other than to the intact skin.

For this, intact skin and damaged skin model were prepared according to the previous study (de Oliveira et al., 2014). Briefly, 15 healthy male standard deviation (SD) rats (weight 180 ∼ 220 g) were shaved on the back and were kept under observation for 24 h to check any damage on the skin appear. In case, any damage was found on skin of any rat, was not involved in study. After 24 h of observation, shaved area on the back of rats was divided into two regions, one for intact and other for damage. To develop damaged region, scalpel was used to make abrasion on the skin with slight bloody appearance, as shown in Supplementary Figure S1. After that, the rats were divided into five groups randomly, for control saline, blank nanoemulgel, EPR nanoemulgel, blank nanoemulsion, and EPR nanoemulsion. The preparations were applied on the both regions at 2 cm × 2 cm area. All the animals were provided with same ad libitum access at 25 °C and 55% humidity room condition.

All the animals were monitored for presence of erythema and edema at 1, 24, 48, and 72 h time interval and the symptoms were scored based on the visibility of symptoms as listed in Supplementary Table S1 and S2 (Jia et al., 2008). Average stimulation score = Σ(sum of erythema scores + sum of edema scores)/number of animals (Al Hanbali et al., 2019).

After completion of 72 h study, the rats were sacrificed by cervical dislocation, and the skin samples from the test area were collected from the center part with area of about 1 cm × 1 cm. The collected samples were preserved in 4% polyformaldehyde at 4 °C. The skins were embedded in paraffin and then sectioned into 5-μm thick sections and stained with H&E dye. The histological changes were imaged with a light microscope (Nikon, Tokyo, Japan).

#### Skin retention and *in vivo* permeation study

The skin retention and *in vivo* permeation of EPR formulations were studied to evaluate the drug permeability through skin. FITC was used as a model dye in the place of EPR this experiment. Different formulations (FITC nanoemulgel, FITC emulgel, and FITC suspension) were prepared (at the concentration of 0.04% FITC w/w) in the same manner as EPR formulations

For *in vivo* study, ICR mice were randomly divided into four groups, and their hair on the back was removed with 8% sodium sulfide 12 h before application. Four groups of mice were applied with FITC solution, FITC nanoemulgel, FITC emulgel, and FITC suspension, respectively. The applied area was approximately maintained at 2 cm × 2 cm. The mice were sacrificed at 1, 4, and 8 h, and the skin was washed with normal saline and 1 cm × 1 cm area was taken out for tissue sections and was observed by fluorescence microscope (Nikon Eclipse C1, Nikon, Tokyo, Japan).

#### Statistical analysis

The results are expressed as mean ± SD, with the data being statistically analyzed for the Student’s *t* test (two groups) and one-way analysis of variance (ANOVA, multiple groups). The differences were considered statistically significant at *p* < .05. Statistical analysis was performed by SPSS 22.0.

## Results and discussion

### Single factor design of experiment

A single factor experimental design was employed to statistically optimize the formulation parameters of EPR nanoemulgel for better stability.

### Effect of the non-aqueous agent

**Figure 3.**
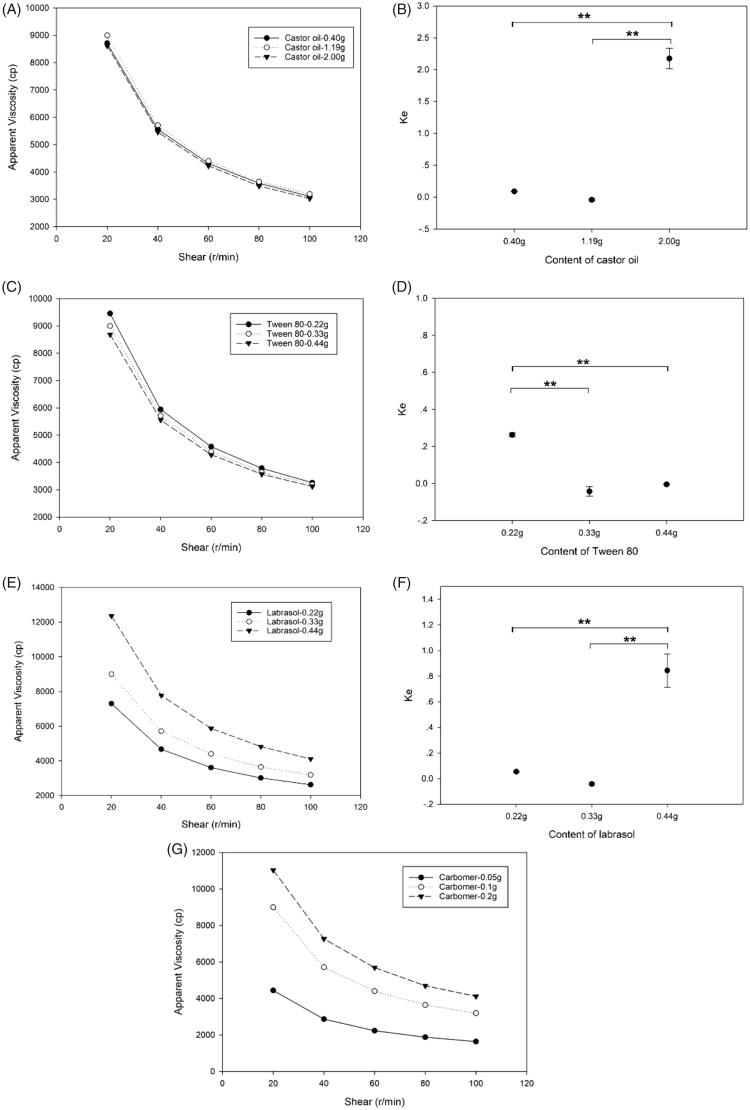
The effect of different compositions on viscosity and *Ke* of EPR formulations. A, C, E and G represented the effects of content of castor oil, Tween 80, Labrasol^®^, and carbomer on the viscosity of EPR nanoemulgel, respectively; B, D, and F represented the *Ke* of EPR nanoemulsion containing different amount of castor oil, Tween 80, and Labrasol^®^, respectively. (**: *p* < .01).

From Figure 3(A,B), it can be seen that the content of castor oil has no significant effect on viscosity of EPR nanoemulgel but has significant effect on *Ke*. The *Ke* values of EPR nanoemulsion which contain 0.40 and 1.19 g castor oil were close to 0, whereas the *Ke* of 2.00 g castor oil group was 2.174, which implied that the nanoemulsion system would be unstable when the content of non-aqueous agent is over a critical value. Therefore, the content of non-aqueous agent, i.e. castor oil selected for this formulation was 1.19 g (10.8% w/w in nanoemulsion).

### Effect of the emulsifier

Figure 3(C,D) depicted the effect of emulsifier’s content on the viscosity of nanoemulgel and stability of nanoemulsion. It was found that the viscosity of nanoemulgel would not be influenced by the content of Tween 80. However, EPR nanoemulsion containing 0.11 and 0.22 g of Tween 80, respectively has higher (*p* < .01) *Ke* values compared to the other two groups, this indicated the formation of weaker bond and can be easily separated into two layers. The reduction of emulsifier’s content leads to the lowering of emulsification effect which further causes easy aggregation of oil droplets. To decrease the skin irritation caused by surfactant, the formulation contains less surfactant was preferred. In this case, the optimized EPR nanoemulsion contains was 0.33 g Tween 80 (3% w/w in nanoemulsion).

### Effect of the co-emulsifier

Figure 3(E) showed the effect the co-emulsifier. As can be seen from figure, the increase in content of Labrasol^®^ from 0.22 to 0.44 g, leads to higher viscosity of EPR nanoemulgel, this may be due to decrease of HLB value, which strengthen the interaction between hydrophilic polymer and oil droplets. The EPR nanoemulsion contains 0.44 g Labrasol^®^ exhibited the worst stability. From the design of experiment, it was found that the optimum quantity of Labrasol^®^ was 0.33 g at which the formulation showed good rheological characteristics and permeability.

### Effect of carbomer on viscosity of nanoemulgel

As can be seen in Figure 3(G), Carbomer has direct effect on the viscosity of nanoemulgel, increasing the concentration of Carbomer from 0.05 to 0.2 g can proportionally increases the viscosity. In general, formulation with higher viscosity is beneficial for during application on skin because it retains on the skin for longer period. However, too high viscosity leads to the inconvenience in use, because it cannot spread sufficiently and also it retards the movement of loaded drugs due to presence of higher cross-linked chains (Jain et al., 2016). In this case, the optimized concentration of Carbomer was fixed to 0.1 g.

### Rheological properties

The results presented in Figure 3 show the relationship between viscosity and shear rate. Here, all the prepared EPR nanoemulgels exhibit non-Newtonian shear-shinning behavior, which originated from the broken of entanglements or physical nodes that are responsible for raising the viscosity of nanoemulgel. Then, the polymer chains realign in the direction of the applied strain, thus lead to the lowering of viscosity (Barradas et al., 2017).

This shear-thinning behavior is suitable in transdermal delivery system for the nanoemulgel, which would not drip on the finger or at the application site. What is more, it makes it easy to spread on the skin surface uniformly.

### Zeta size analysis and zeta potential

The zeta size analysis showed that the optimized EPR nanoemulsion has average droplet size of 327.43 ± 10.35 nm at 25 °C with PDI 0.183. In addition, the average zeta potential of optimized EPR nanoemulsion was –29.10 ± 1.27 mV, which indicates that the nanoemulsion was stable. At the same time, emulsion that prepared for emulgel has average droplet size of 1154.54 ± 296.08 nm with PDI 0.332 and its zeta potential was –44.06 ± 1.28 mV (Supplementary Figure S2).

### Morphology analysis

**Figure 4.**
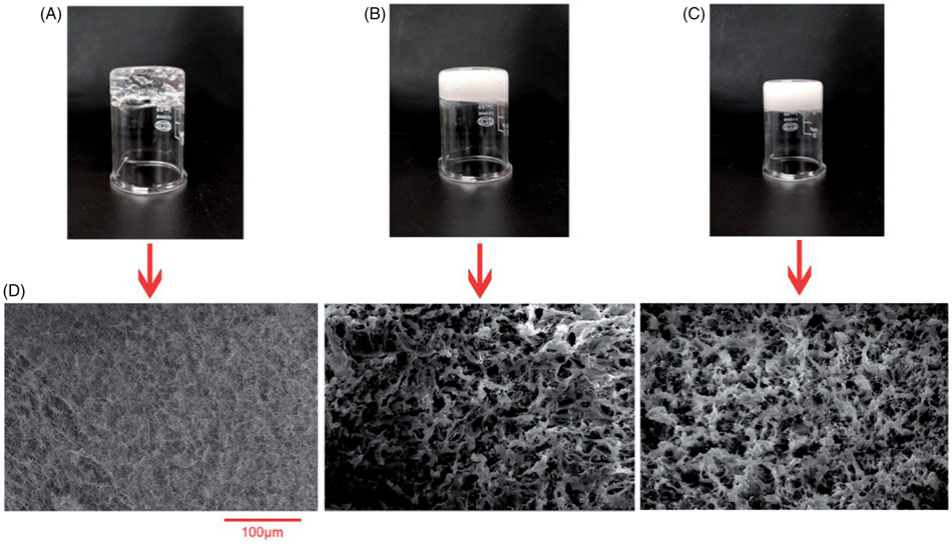
The appearance of 1% carbomer hydrogel (A), blank nanoemulgel (B), EPR nanoemulgel (C), and their SEM images (D).

The physical appearance and FE-SEM images of 1% carbomer hydrogel, blank nanoemulgel and EPR nanoemulgel were shown in Figure 4. As shown in figure, 1% carbomer hydrogel is transparent while blank nanoemulgel and EPR nanoemulgel are white semisolid due to the incorporation of nanoemulsion. All the formulations were homogeneous, stable, and viscous (Figure 4(A–C)).

The SEM images of 1% carbomer hydrogel looks like a tiny mesh pores whereas the images of blank nanoemulgel and EPR nanoemulgel exhibited interconnected pores with random size distribution. This porous structure provides sufficient space for high drug loading, movement of drug throughout and enhance the drug release rate.

### Bioadhension of different EPR emulgels

The concentration of carbomer is directly proportional with the adhesion property of the EPR nanoemulgel as can be seen from the Table 1. The ‘*f*’ value of nanoemugel B and C are significantly differ (*p* < .01) from nanoemugel A. Mechanism of mucoadhesion have been explained by several theories such as electronic theory, wetting theory, and so on. Bioadhesion is a critical factor in transdermal drug delivery systems (TDDS), since the drug absorption is related to the drug partition between the TDDS. Generally speaking, a constant TDDS/skin contact over the application period allows a consistent drug delivery and absorption (Banerjee et al., 2014). Here, the bioadhesion of EPR nanoemulsion was tested as control, and it exhibits almost no bioadhesion to the skin so that it could not be suitable for long-term treatment.

**Table 1.** The bioadhesion of different EPR nanoemulgels (g/cm^2^, mean ± SD).

Samples	*f*	RSD (%)
Nanoemulsion	–	–
Nanoemulgel A	3.38 ± 0.07	2.1
Nanoemulgel B	5.13 ± 0.08**	1.6
Nanoemulgel C	7.12 ± 0.48**	6.7

### *In vitro* permeation of EPR nanoemulgel

*In vitro* experiments on transdermal penetration were carried out to investigate the effect of emulgel and nanoemulgel on transdermal absorption of EPR. The results showed that EPR permeated through the skin at a constant rate and the diffusion behaviors were in accordance with zero-order kinetics (Supplementary Figure S3). The cumulative permeated amount of EPR from different formulations is listed in Table 2.

**Table 2.** Transdermal penetration parameters of different EPR formulations (*n* = 3, mean ± SD).

Formulation	Equation	*P*_app_ × 10^9^ (cm s^–1^)	*J*_ss_ (μg cm^–2^ h^–1^)
EPR suspension	*Q_n_*/*A* = 0.14*t* + 0.071, *r*^2^ = 0.951	7.52 ± 1.95	0.14 ± 0.04
EPR emulgel	*Q_n_*/*A* = 0.78*t* – 1.43, *r*^2^ = 0.966	43.14 ± 8.05*	0.78 ± 0.14*
EPR nanoemulgel	*Q_n_*/*A* = 1.13*t* – 1.99, *r*^2^ = 0.969	62.87 ± 10.97*^#^	1.13 ± 0.20*^#^

Comparatively, nanoemulgel and emulgel significantly enhance (*p* < .01) the permeability of EPR by 8.07 fold and 5.57 fold compared to suspension, which indicates that the nanoemulgel has better absorption than suspension as a result of multiple factors including skin hydration, the loose of stratum corneum lipid bilayers by surfactant and the penetration of oil droplets through stratum corneum (Ajazuddin et al., 2013; Sengupta & Chatterjee, 2017). Additionally, the permeation rate of nanoemulgel was significantly faster than emulgel by 1.45 fold due to the larger surface for transfer (Hu et al., 2019).

### Cytotoxicity

The cytotoxicity of EPR formulations were evaluated by MTT assay using L929 fibroblasts cell line. The results shown in Supplementary Figure S4 revealed that both EPR suspension and nanoemulgel possess no obvious cytotoxicity at the concentration ranges from 1 μg/ml to 10 mg/ml and there was no significant difference (*p* > .05) between the two groups.

According to the available information in the *FDA Inactive Ingredient Search for Approved Drug Products*, the maximum amount of Tween 80 in gel is 8.5% w/w, Caprylocaproyl Macrogolglycerides (Labrasol^®^) in cream 7.5% w/w, castor oil in ointment is 14.9% w/w and carbomer 940 in gel is 1.1% w/w for topical use. It suggested that EPR nanoemulgel consisting of 0.27% w/w Tween 80, 0.27% w/w Labrasol, 0.98% w/w castor oil, and 1% w/w carbomer 940 can be a promising and safe candidate for transdermal application.

### Skin irritation test

The safety of the EPR nanoemulgel was further evaluated to ensure that it has low skin toxicity and causes negligible irritation to the skin.

The average skin stimulation scores of Supplementary Table S3 indicated that there is no irritation observed in blank nanoemulsion, blank nanoemulgel, EPR nanoemulsion, and EPR nanoemulgel on both intact and damaged skin. Moreover, in order to eliminate the limitation of visual acuity, the histological analysis is preferable to observe if there is any possible changes on the skin.

**Figure 5.**
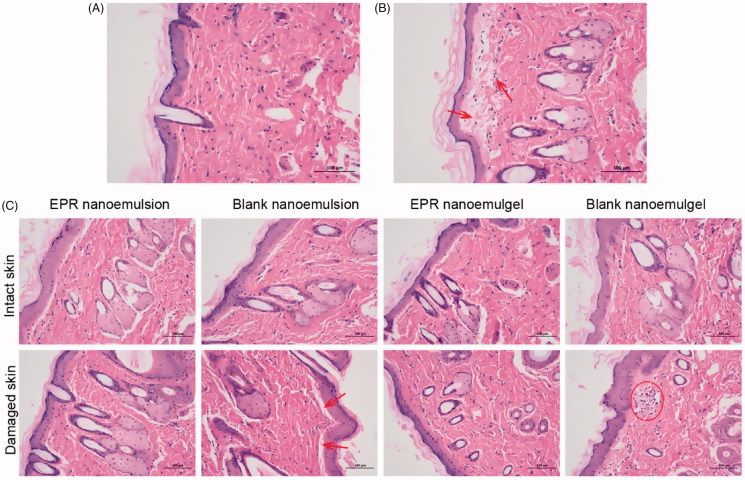
H&E staining of intact skin (A) and damaged skin (B) applied with normal saline, intact skin and damaged skin applied with different formulations (C) for 72 h (200×). Arrows indicate the edema and lymphocytes.

The histopathological results (Figure 5(A)) indicated that the intact skin applied with normal saline has no inflammation, the epithelial cells were arranged neatly, the collagen fibers in the dermis were arranged regularly, and the accessory organs such as the hair follicle sebaceous glands were observed without any damages. In the damaged skin group, mild edema was observed in the upper part of the dermis, and a single lymphocyte infiltration was observed (Figure 5(B)).

As for intact skin applied with different EPR formulations, no obvious inflammation was observed, indicating that the formulations were not irritating to the intact skin of the rats (Figure 5(C)). A small amount of inflammatory cell infiltrations was found in the skin of control groups, while the damaged skin administered with EPR nanoemulsion and the EPR nanoemulgel was intact and exhibited no significant inflammation. It was reported that avermectin and its derivative ivermectin exerted anti-inflammation effect (Ci et al., 2009; Yan et al., 2011). As the EPR is the derivative of avermectin, it may also have the anti-inflammatory effect. The overall results indicated that the EPR nanoemulgel were within the limit of the skin tolerance and safe to use in transdermal applications.

### *In vivo* skin retention and permeation study

**Figure 6.**
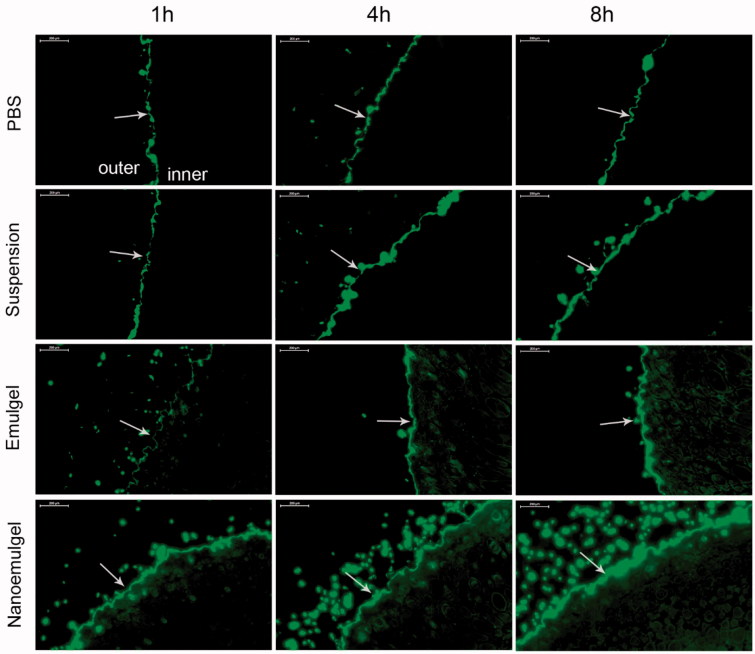
Fluorescence microscopy of mice skin applied with different FITC formulations. Arrows indicate the stratum corneum of the skin.

The result of *in vivo* skin retention and permeation study is shown in Figure 6. As explained in method, FITC was used as a model drug to replace EPR to prepare FITC nanosemulgel and emulgel. The FITC solution was kept as control group. FITC concentration was maintained same in all samples and was applied on the skin of animals. After 8 h, skins were imaged under fluorescence microscope; the obtained images showed almost no penetration of FITC into the skin of animals applied with FITC suspension. This may be due to lower contact duration with applied skin because of weaker bioadhesion property of suspension.

Compared to the liquid preparation, FITC emulgel and nanoemulgel possess better bioadhesion property and prolonged retention time. After application of 4 h, penetration of FITC was seen into the dermis region of skin and was more prominent at 8 h, which indicates emulgel can promote the transdermal delivery of the drug. Similarly, in the nanoemulgel group, drug loaded into nano oil droplets have larger surface area to move around and have high tendency to diffuse out from the matrix. As can be seen from the images of FITC nanoemulgel, the penetration of FITC started just after 1 h application and gradually increases with the passes of time. Comparing 8 h images from all groups, it is clear that the nanoemulgels have high capacity to deliver loaded drugs into the dermal region of the skin in comparison to emulgel and liquid preparations. From this, it can be concluded that EPR loaded nanoemugel can retain for longer period on the skin and can release sufficient amount of drug to provide prolonged therapeutic local action.

## Conclusion

This study reported the synthesis and characterization of nanoemulgel as a promising drug carrier to load and deliver a lipophilic antiparasitic drug, EPR. *In vitro* cytotoxicity study and skin irritation test revealed that the concentration of pharmaceutical excipients used in the formulation is well under tolerate amount and safe. Due to good bioadhensive property compared to liquid medications, this nanoemulgel retain for prolonged period at applied area. The presence of hydrogel matrix and O/W emulsion system in nanoemulgel, made it a unique TDDS. O/W emulsion system provided sufficient non-aqueous surface to load hydrophilic drug and the hydrogel matrix help to trap nanosized drug loaded droplets for prolonged period. In conclusion, this EPR loaded nanoemulgel is safe and promising TDDS for the treatment of endo-and ectoparasites.

## Supplementary Material

Supplemental Material
