# Supplementary material for: Eprinomectin nanoemulgel for transdermal delivery against endoparasites and ectoparasites: preparation, in vitro and in vivo evaluation
Source: Drug Deliv. 2019 Nov 18;26(1):1104–14. doi: 10.1080/10717544.2019.1682720 (PMC6882498; doi:10.1080/10717544.2019.1682720)
Supplement: Supplemental Material [file IDRD_A_1682720_SM7544.docx]

Supplemental material

**Eprinomectin nanoemulgel for transdermal delivery against endo- and ectoparasites: preparation, *in vitro* and *in vivo* evaluation**

Yujuan Mao^1#^, Xiaolan Chen^1#^, Bohui Xu^2^, Yan Shen^3^, Zixuan Ye^3^, Birendra Chaurasiya^2^, Li Liu^1^, Yi Li^4^, Xiaoling Xing^1*^, Daquan Chen^4*^

^1^ Jiangsu Animal Husbandry and Veterinary College, Taizhou, China

^2^School of Pharmacy, Nantong University, No.19 Qixiu Road, Nantong 226001, PR China

^3^Department of Pharmaceutics, School of Pharmacy, China Pharmaceutical University, Nanjing, China

^4^School of Pharmacy, Yantai University, Yantai, China

^#^These authors contributed equally to this work.

*Corresponding author: Xiaoling Xing. Tel: +86 0 523 86156928. E-mail: [jstzxln@sina.com;](mailto:jstzxln@sina.com;) Daquan Chen. Tel: 0535-6706021. Email: cdq1981@126.com


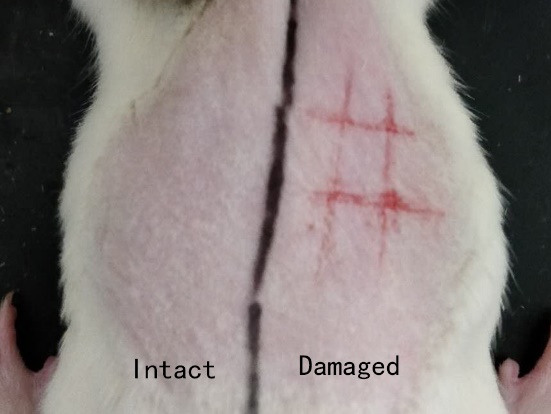


**Figure S1.** Intact skin and damaged skin of rats.

**Table S1.** The score of erythema according to symptoms

| Symptoms | Score |
| --- | --- |
| No erythema | 0 |
| Mild erythema (barely seen) | 1 |
| Visible | 2 |
| Severe erythema | 3 |
| Dark red erythema with scab | 4 |

**Table S2.** The score of edema according to symptoms

| Symptoms | Score |
| --- | --- |
| No edema | 0 |
| Mild edema (barely seen) | 1 |
| Bump with clear boundary | 2 |
| Bump up about 1cm | 3 |
| Bump up>1 cm, range increase | 4 |


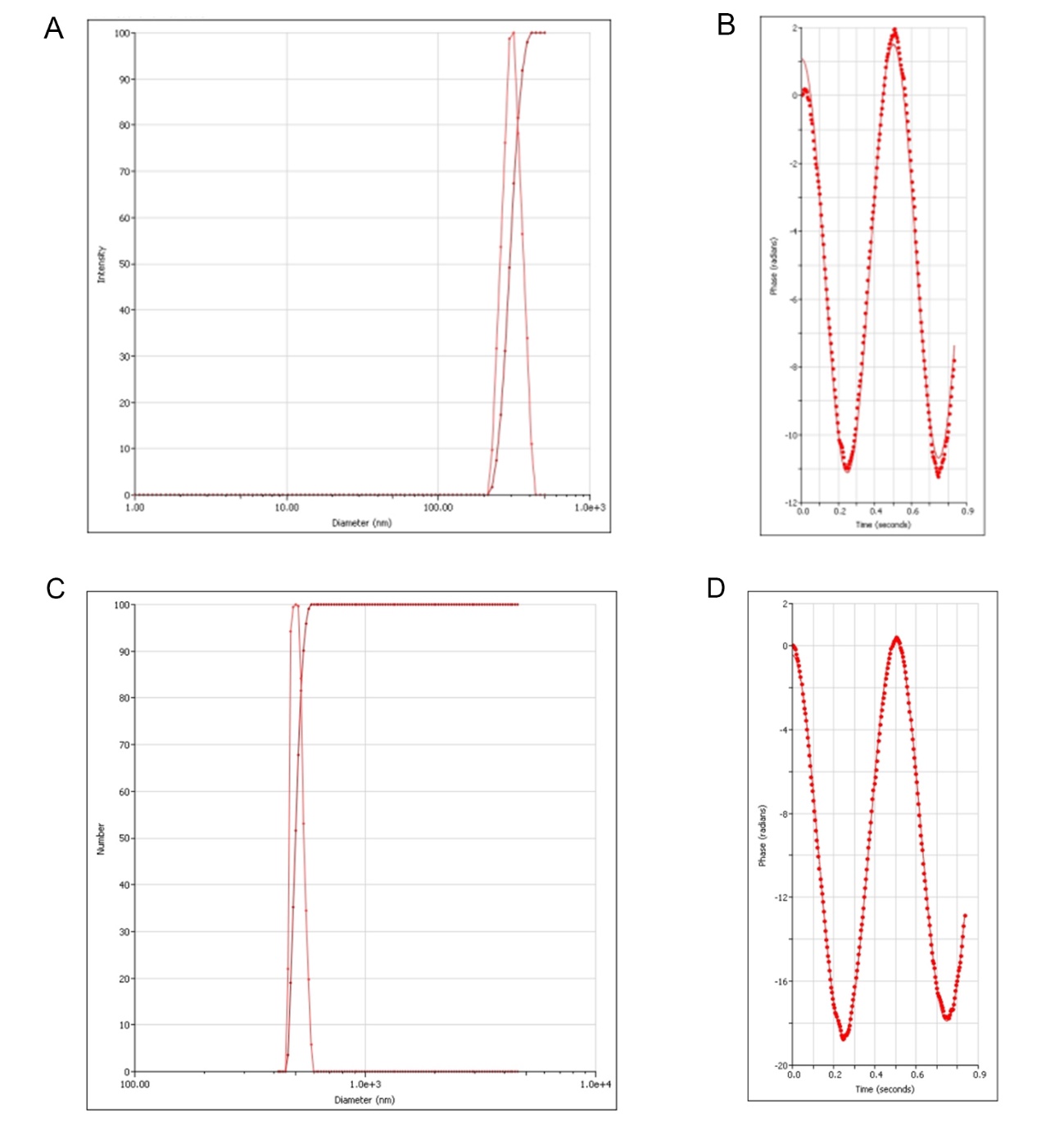


**Figure S2.** Zeta size and zeta potential of optimized EPR nanoemulsion (A & B) and EPR emulsion (C & D).


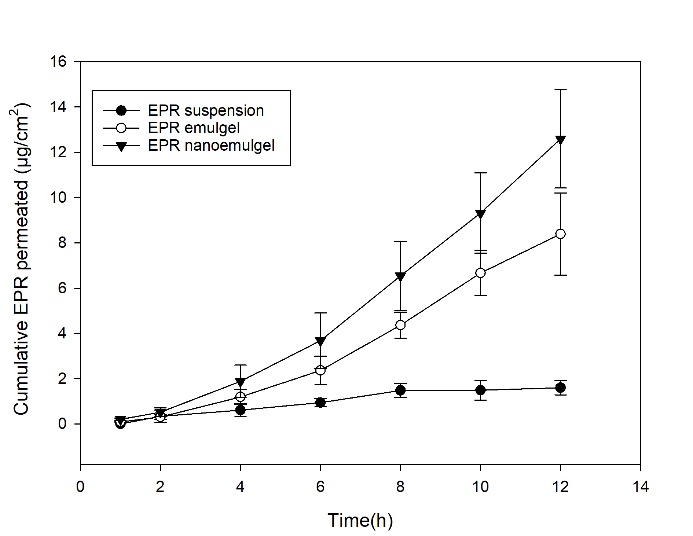


**Figure S3.** The cumulative transdermal permeation profile of different EPR formulations (n=3, mean±SD)


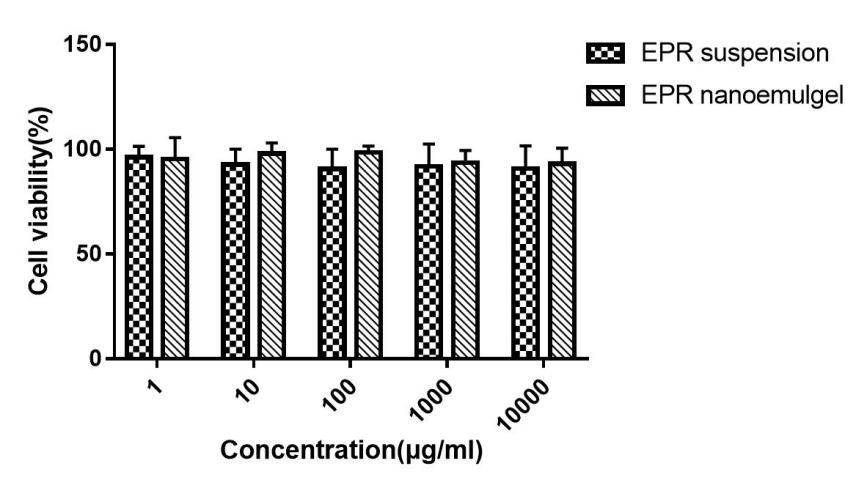


**Figure S4.** Cell viability (%) exposed to EPR suspension and EPR nanoemulgel at different concentration.

**Table S3.** Average skin stimulation scores of different group (n=3)

| Group | Intact/damaged skin | Scores | | | |
| --- | --- | --- | --- | --- | --- |
|  |  | 1(h) | 24(h) | 48(h) | 72(h) |
| Normal saline | Intact | 0 | 0 | 0 | 0 |
|  | Damaged | 0 | 0 | 0 | 0 |
| EPR nanoemulgel | Intact | 0 | 0 | 0 | 0 |
|  | Damaged | 0 | 0 | 0 | 0 |
| Blank nanoemulgel | Intact | 0 | 0 | 0 | 0 |
|  | Damaged | 0 | 0 | 0 | 0 |
| EPR nanoemulsion | Intact | 0 | 0 | 0 | 0 |
|  | Damaged | 0 | 0 | 0 | 0 |
| Blank nanoemulsion | Intact | 0 | 0 | 0 | 0 |
|  | Damaged | 0 | 0 | 0 | 0 |
